# Takotsubo Cardiomyopathy after Spinal Anesthesia for a Minimally Invasive Urologic Procedure

**DOI:** 10.1155/2017/8641641

**Published:** 2017-06-13

**Authors:** Emmanuel Lilitsis, Despina Dermitzaki, Georgios Avgenakis, Ioannis Heretis, Charalampos Mpelantis, Charalampos Mamoulakis

**Affiliations:** ^1^Department of Anesthesiology, University General Hospital of Heraklion, University of Crete, Medical School, Heraklion, Crete, Greece; ^2^Department of Urology, University General Hospital of Heraklion, University of Crete, Medical School, Heraklion, Crete, Greece

## Abstract

We present the case of a patient who suffered from Takotsubo cardiomyopathy (TCM) immediately after the initiation of subarachnoid anesthesia for a minimally invasive urologic procedure (tension-free vaginal tape (TVT) surgery for stress urine incontinence). TCM mimics acute coronary syndrome and is caused by an exaggerated sympathetic reaction to significant emotional or physical stress. Our patient suffered from chest pain, palpitations, dyspnea, and hemodynamic instability immediately following subarachnoid anesthesia and later in the postanesthesia care unit. Blood troponin was elevated and new electrocardiographic changes appeared indicative of cardiac ischemia. Cardiac ultrasound indicated left ventricular apical akinesia and ballooning with severely affected contractility. The patient was admitted to coronary intensive care for the proper care and finally was discharged. TCM was attributed to high emotional preoperative stress for which no premedication had been administered to the patient. In conclusion, adequate premedication and anxiety management are not only a measure to alleviate psychological stress of surgical patients, but, more importantly, an imperative mean to suppress sympathetic nerve system response and its cardiovascular consequences.

## 1. Introduction 

Takotsubo cardiomyopathy (TCM) or stress-induced cardiomyopathy or “broken heart syndrome” is a transient cardiac syndrome that was first described in Japan in 1990 by Sato et al. [1]. The Japanese word* Takotsubo* translates to “octopus trap,” a pot with a wide base and narrow top, which left ventricle resembles during systole. Although considered rare diagnosis, TCM has been identified in 2% of patients presenting with acute coronary syndrome [2]. Patients at high risk to develop the syndrome are considered to be postmenopausal women under severe emotional or psychological stress. TCM mimics acute coronary syndrome and is usually presented with chest pain, ST-segment elevation on electrocardiogram (ECG), and elevated cardiac enzyme levels consistent with a myocardial infarction. However, when the patient undergoes cardiac angiography, left ventricular apical ballooning is present and there is no significant coronary artery stenosis. At time of presentation, cardiogenic shock and malignant arrhythmias may be present [3]. Although the exact etiology is still unknown, the syndrome appears to result from an exaggerated sympathetic “crisis” triggered by significant emotional or physical stress, leading to catecholaminergic storm and myocardial stunning [4]. There are numerous cases published reporting perioperative cardiomyopathy, mostly related either to surgery under general anesthesia or to administration of vasoactive drugs, such as epinephrine, ephedrine, or dobutamine [5]. Preoperative anxiety and anticipation of surgery are also included in the so-called stress response to anesthesia and surgery [6]. We present the case of a patient who suffered from stress-induced cardiomyopathy with severe left ventricular dysfunction immediately after the initiation of subarachnoid anesthesia for a minimally invasive urologic procedure.

## 2. Case Presentation

A 46-year-old, premenopausal Caucasian woman with body mass index of 24 kg/m^2^ was scheduled for a minimally invasive urologic procedure (tension-free vaginal tape (TVT) surgery for stress urine incontinence). Preoperative evaluation revealed an unusually nervous patient quoting very unpleasant experience regarding prior uneventful operations (appendectomy in childhood; caesarian section twice) and postulating not to be admitted before the day of surgery. Her medical history included Hashimoto's disease adequately controlled by thyroxine replacement therapy (ASA class II). Cardiovascular and respiratory clinical examination was normal on admission (blood pressure (BP): 116/57 mmHg, heart rate (HR): 58 beats per minute, and SpO_2_ 99%). Preoperative 12-lead ECG, chest X-ray, and routine laboratory tests were normal. Informed consent was obtained for subarachnoid anesthesia, the standard for TVT surgery. The patient asked to be discharged the same day, if possible. Premedication, although routinely used for ASA II patients, was withheld because there was a concern about delay from hospital discharge, which was the patient's primary wish.

Upon arrival to the operating room, vital signs recordings were BP: 110/65 mmHg, HR: 75 bpm, and SpO_2_ 98% (room air). Although anxious, she was cooperative. Successful subarachnoid anesthesia was performed with 10 mg bupivacaine heavy 5% and 20 *μ*g fentanyl. Immediately after the subarachnoid injection, the patient complained of chest pain, palpitations, dyspnea, and nausea. BP dropped to 65/43 mmHg and HR increased to 94 bpm, while several supraventricular ectopic beats appeared. SpO_2_ was 98% on O_2_ nasal cannula 2 L/min. A bolus of 10 mg of ephedrine was administered along with a 250 ml bolus crystalloid fluids and BP increased slightly to 75/50 mmHg. The patient continued to complain of dyspnea and chest pain. A second bolus of 15 mg of ephedrine was administered along with 2 mg of midazolam for anxiety relief. BP and HR reached 103/68 mmHg and 110 bpm, respectively. She stopped complaining and remained calm and stable thereafter. Nevertheless, persistent hypotension was recorded throughout the operation (systolic BP: 90–100 mmHg, HR: 90–110 bpm), which was attributed to the anesthetic technique and the procedure was uneventfully completed within 30 minutes.

After surgery, the patient was transferred to the postanesthesia care unit for complete recovery from motor and sensory blockade. Five minutes later, she complained of chest pain again and she became hypotensive (BP: 72/59 mmHg), dyspneic, and tachypneic (SpO_2_ 88% on nasal cannula). Phenylephrine infusion was started at 400 *μ*g/h and nasal cannula was switched to a 50% Venturi mask with normalization of BP and SpO_2_. Auscultation of lungs and heart revealed no pathologic sounds. A new 12-lead ECG indicated sinus rhythm with no ST elevation or depression and—not preexisting—poor progress of R waves on precordial leads and negative T wave on leads I and AVL. The new chest X-ray was normal. Arterial blood gases showed PO_2_ 78 mmHg, PCO_2_ 27 mmHg, and pH 7.43 on 50% Venturi mask. Biochemical tests were normal apart from troponin which was elevated 9.407 ng/ml (normal < 0.04) and D-dimmers 1245 ng/ml (probably expected postoperatively). Since there was no residual motor or sensory blockade but the patient was still on phenylephrine, a spiral computed tomography was performed. The possibility of pulmonary embolism was excluded but a suspicion of interstitial/alveolar edema was set. A cardiac ultrasound detected left ventricular apical akinesia and ballooning with severely affected contractility sparing basic parts and ejection fraction ~20%. The patient was transferred to the coronary intensive care unit for further assessment. Emergency coronary angiography revealed no indication of stenosis. On left ventriculography, there were decreased apical contractility and balloon shape with contraction of basic parts. Ejection fraction was approximately 25%.

A diagnosis of Takotsubo cardiomyopathy was made according to Mayo Clinic diagnostic criteria [7]. For the next day, she remained severely dyspneic and unable to sustain normal SpO_2_ with supplemental O_2_. New chest X-ray was compatible with acute pulmonary edema. She received noninvasive mechanical ventilation in order to decrease preload and afterload of the left ventricle and to alleviate work of breathing. Drug therapy was initiated (furosemide 10 mg × 3, carvedilol 6,25 mg × 2, and bromazepam 1.5 mg × 3). A rhythm Holter was applied with no complex ventricular arrhythmogenesis. Within the following six days, troponin levels normalized as well as left ventricular systolic performance (ejection fraction reached 50%) on consecutive ultrasounds. The patient was transferred to the cardiology department and was discharged in good physical status seven days later with follow-up instructions.

## 3. Discussion

A case of TCM immediately after initiation of subarachnoid anesthesia for a minimally invasive urologic procedure is presented. Stress-induced cardiomyopathy has been previously reported to happen throughout the perioperative period as a result of an exaggerated adrenergic response to increased perioperative physical and emotional stress. Both increased circulating catecholamines and sympathetic outflow in cardiac nerve endings result in impaired myocardial perfusion, myocyte injury, and left ventricular outflow tract obstruction [3]. Regarding perioperative period, TCM has been identified immediately prior or during the induction of general anesthesia [8, 9], as well as intra- and postoperatively [10–14]. Perioperative pain and anxiety, light depth of anesthesia, and increased stress response to surgery have been identified as factors responsible for stress-induced cardiomyopathy. Regarding regional anesthesia, TCM has already been reported 15 min following subarachnoid anesthesia for caesarean section and our case is the second one in series [15].

These two case reports shed light on regional anesthesia as not “stress-free” anesthetic since an awake patient cannot always tolerate the emotional stress. Takotsubo cardiomyopathy in our patient was attributed to exaggerated preoperative anxiety. The latter should not be regarded as an innocent emotional state of surgical patients with minimal effect on actual physical status. Possibly, deteriorating mechanisms for the development of cardiomyopathy were the acute decrease of pre- and afterload of left ventricle and the stressful feeling of rapid ascending level of paralysis, in combination with the psychological background of the patient.

Premedication is no longer considered to prolong hospitalization for one-day adult surgery and such patients should not be denied anxiolytics [16]. It is well known that preoperative anxiety and catastrophizing are well correlated not only with the intensity of acute postsurgical pain but also with the development of chronic pain [17, 18]. Emotional stress consists also of an independent factor for arrhythmias and has been implicated for stress-related sudden cardiac death [19, 20]. Proper anxiolytic premedication before surgery also results in lower incidence of surgical site infection even up to 30 days after surgery due to decreased stress response [21]. The application of a proper protocol preoperatively, both pharmacological and nonpharmacological, including patient's training and behaviour modification, could affect outcome and even hospital length of stay [22]. There is no consensus regarding the most appropriate anesthetic management for patients with a prior history of stress-induced cardiomyopathy. Although relied on weak evidence, regional anesthesia is considered as the safest and most appropriate anesthetic technique for those patients [23].

## 4. Conclusion 

A case of a patient who suffered stress-induced cardiomyopathy following subarachnoid anesthesia was presented. It is assumed that exaggerated preoperative anxiety, for which no premedication was given, caused a catecholaminergic storm state to the patient. Regional anesthesia, under certain circumstances, can be a quite harmful situation inducing an even higher adrenergic response. Management of preoperative anxiety, as an integral part of anesthetic practice, should target not only the patient's psychological comfort, but, most importantly, the prevention of possible cardiovascular and other complications related to it.
